# High-Density Lipoprotein in Patients with Diabetic Kidney Disease: Friend or Foe?

**DOI:** 10.3390/ijms26041683

**Published:** 2025-02-16

**Authors:** Ke Liu, Mark E. Cooper, Zhonglin Chai, Fang Liu

**Affiliations:** 1 Department of Nephrology, West China Hospital of Sichuan University, Chengdu 610041, China; liukee@stu.scu.edu.cn; 2 Laboratory of Diabetic Kidney Disease, Kidney Research Institute, Department of Nephrology, West China Hospital, Sichuan University, Chengdu 610041, China; 3 Department of Diabetes, School of Translational Medicine, Monash University, Melbourne, VIC 3004, Australia; mark.cooper@monash.edu

**Keywords:** high-density lipoprotein, cholesterol, type 2 diabetes, diabetic kidney disease, chronic kidney disease

## Abstract

High-density lipoprotein (HDL) exhibits multiple metabolic protective functions, such as facilitating cellular cholesterol efflux, antioxidant, anti-inflammatory, anti-apoptotic and anti-thrombotic properties, showing antidiabetic and renoprotective potential. Diabetic kidney disease (DKD) is considered to be associated with high-density lipoprotein cholesterol (HDL-C). The hyperglycemic environment, non-enzymatic glycosylation, carbamylation, oxidative stress and systemic inflammation can cause changes in the quantity and quality of HDL, resulting in reduced HDL levels and abnormal function. Dysfunctional HDL can also have a negative impact on pancreatic β cells and kidney cells, leading to the progression of DKD. Based on these findings, new HDL-related DKD risk predictors have gradually been proposed. Interventions aiming to improve HDL levels and function, such as infusion of recombinant HDL (rHDL) or lipid-poor apolipoprotein A-I (apoA-I), can significantly improve glycemic control and also show renal protective effects. However, recent studies have revealed a U-shaped relationship between HDL-C levels and DKD, and the loss of protective properties of high levels of HDL may be related to changes in composition and the deposition of dysfunctional particles that exacerbate damage. Further research is needed to fully elucidate the complex role of HDL in DKD. Given the important role of HDL in metabolic health, developing HDL-based therapies that augment HDL function, rather than simply increasing its level, is a critical step in managing the development and progression of DKD.

## 1. Introduction

In 2022, 828 million adults (aged 20–79 years) worldwide were estimated to be living with diabetes. The number of adult diabetic patients in China is about 148 million, accounting for 18% of the total number of adults with diabetes in the world [1]. Management of diabetes and its complications, such as cardiovascular disease, retinopathy, neuropathy and kidney disease, is a major public health priority and is projected to account for over 15% of total global healthcare expenditures by 2040 [2]. Diabetic kidney disease (DKD) is one of the major diabetic complications affecting 40–50% of type 2 diabetes (T2D) patients and is a major challenge for the healthcare system [3]. Diabetes-associated chronic kidney disease (CKD) often progresses to end-stage renal disease (ESRD) in association with multiple complications, often vascular in nature. The number of patients with diabetic ESRD requiring dialysis treatment or kidney transplantation is rising, with diabetes-associated ESRD accounting for >50% of newly diagnosed cases of ESRD in some countries [3,4]. Effective management of the early stages of DKD can significantly delay the onset of end-stage renal disease [5]. Therefore, the discovery of biological predictors and risk factors for DKD should lead to the early identification of high-risk individuals and contribute to approaches to prevent diabetic complications and slow down progression.

Hyperglycemia and hypertension are established modifiable factors associated with increased risk of DKD progression, with the role of dyslipidemia remaining controversial [4,6,7,8]. Studies have suggested that changes in lipid metabolism and lipid deposition in the kidneys may play a crucial role in the worsening of DKD; however, the actual impact of dyslipidemia on DKD is not fully understood. Although elevated low-density lipoprotein cholesterol (LDL-C) levels are linked to kidney disease progression, the direct impact of high-density lipoprotein cholesterol (HDL-C) levels on kidney disease, particularly in T2D patients, remains poorly understood due to limited evidence being available [8,9]. The role of high-density lipoprotein (HDL) and its major apolipoprotein component apolipoprotein A-I (apoA-I) in cardiovascular protection has been widely studied and recognized [10]. In addition to cardioprotection, HDL and apoA-I also showed glucose-lowering properties [11]. Studies have shown that apoA-I can improve glycemic control by increasing insulin production and secretion, reflecting improved pancreatic β-cell function [12,13]. Moreover, lower HDL-C levels are recognized as a significant and independent predictor of DKD [14]. Recent studies have demonstrated a U-shaped association between HDL-C levels and kidney disease outcomes, which means that higher levels of HDL-C within a certain range are protective, but at even higher levels, HDL-C loses its kidney-protective effects [15]. Thus, the complex relationship between HDL-C and clinical outcomes, including incident ESRD and all-cause mortality, in patients with T2D and CKD needs to be investigated more comprehensively, and the impacts of HDL subfractions on the progression of DKD need to be more robustly examined. From the therapeutic point of view, drugs that increase HDL and apoA-I levels appear to prevent and reverse diabetes and its complications [11]. Elucidation of the mechanisms to explain the actions of these drugs should be able to help with the identification of new specific therapeutic targets to facilitate the development of novel and optimized therapeutic strategies. Indeed, it is likely that the new therapeutic strategies will focus more on improving HDL function rather than just increasing its concentration.

This review examines the function of HDL and the regulation of HDL production and metabolism in physiology. The alteration of HDL in the diabetic state and the possible protective and deleterious effects of HDL in DKD are analyzed. The current understanding of HDL and experimental findings are summarized in order to provide novel research perspectives for future endeavors to optimize management of diabetes and DKD.

## 2. Structure and Function of High-Density Lipoproteins

### 2.1. HDL Production and Reverse Cholesterol Transport 

**Figure 1:**
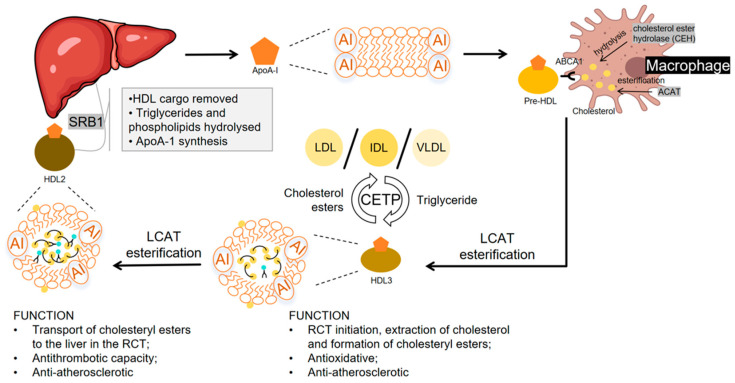
Production and metabolism of HDL. Nascent high-density lipoprotein (HDL) forms in the circulation as apolipoprotein A-I (apoA-I), synthesized by the liver, acquiring phospholipids and cholesterol. Interaction with ATP-binding cassette sub-family A member 1 (ABCA1) on lipid-laden cells activates cholesterol ester hydrolase (CEH), releasing free cholesterol, which is transferred to HDL and re-esterified by phosphatidylcholine–cholesterol acyltransferase (LCAT), converting discoidal HDL3 into cholesterol ester-rich spherical HDL2. Acyl-CoA: cholesterol acyltransferase 1 (ACAT-1) limits cholesterol uptake by promoting intracellular esterification, while cholesterol ester transfer protein (CETP) facilitates the cholesterol ester exchange between HDL and intermediate-density lipoprotein (IDL)/low-density lipoprotein (LDL). In the liver, HDL2 binds scavenger receptor class B type 1 (SR-BI), delivering cholesteryl esters, while hepatic lipase (HL) hydrolyzes triglycerides and phospholipids. Lipid-depleted HDL then reenters the circulation, perpetuating reverse cholesterol transport.

One of the core functions of HDL is to facilitate reverse cholesterol transport, which is the extraction of cholesterol from peripheral tissues, carrying it into the plasma, and delivery to the liver for metabolism and subsequent excretion [16]. HDL formation begins in the liver and small intestine, where apoA-I, a main structural protein component of HDL, is synthesized and receives cholesterol and phospholipids from variable cells (especially macrophages, adipocytes, skin fibroblasts and bone cells) mediated by Adenosine triphosphate (ATP)-binding cassette sub-family A member 1 (ABCA1)/ATP-binding cassette sub-family G member 1(ABCG1) to form the lipid-containing nascent pre(β)-HDL. Cholesterol is esterified by the enzyme phosphatidylcholine–cholesterol acyltransferase (LCAT), resulting in the formation of cholesterol esters. These esterified cholesterols are then incorporated into the core of HDL particles and convert lipid-poor HDL3 into cholesterol ester-rich HDL2, marking the transition from discoidal HDL to mature globular HDL [17]. Mature HDL, also known as α-HDL, reflecting its electrophoretic mobility change from preβ to α, is spherical in shape, with cholesteryl esters, along with triglycerides, forming the hydrophobic core, and phospholipids, free cholesterol, and apolipoproteins forming the hydrophilic shell of the HDL particle. Each mature HDL particle contains a large amount of phospholipids, cholesteryl esters, free cholesterol, triglycerides, and a diverse array of proteins, including apolipoproteins and enzymes. The proportions and types of these components collectively determine the biological properties and functions of HDL [18,19]. In the circulation, some cholesteryl esters of HDL can be replaced with triglycerides from other lipoproteins such as IDL and LDL, through a process facilitated by cholesteryl ester transfer protein (CETP) [20]. In the liver, cholesteryl ester-rich HDL binds to scavenger receptor class B type 1 (SR-BI), facilitating cholesteryl ester uptake, while hepatic lipase (HL) hydrolyzes triglycerides and phospholipids. The depleted HDL then reenters the circulation, initiating another reverse cholesterol transport cycle [16,21] (Figure 1).

### 2.2. Antioxidant Capability 

HDL is known to have antioxidant properties, playing an important role in maintaining cardiovascular health by reducing oxidative stress. HDL particles carry a variety of antioxidant enzymes such as paraoxonase 1 (PON1), LCAT, platelet-activating factor acetylhydrolase (PAF-AH), and lipoprotein-associated phospholipase A2 (LpPLA2), which can reduce lipid oxidation and/or degrade lipid hydroperoxides, thereby exerting its antioxidant effects [22,23,24]. ApoA-I, a major protein component of HDL, binds and removes lipid hydroperoxides from low-density lipoprotein (LDL) in vitro and in vivo, reducing cholesteryl ester and phosphatidylcholine hydroperoxides by converting them into inactive forms via methionine oxidation [18,25,26]. The antioxidant activity of HDL is also reflected in the inhibition of reactive oxygen species (ROS) production and maintenance of endothelial nitric oxide synthase (eNOS) dimer levels and eNOS activity mediated by ABCG1 [27,28]. The antioxidant capacity of HDL is associated with its ability to selectively eliminate HDL-bound lipid hydroperoxides and hydroxides via SR-BI receptors on the surface of hepatocytes [29,30]. In addition, HDL and apoA-I can reduce macrophage-induced oxidative stress by downregulating nicotinamide adenine dinucleotide phosphate (NADPH) oxidase 2 (NOX2) expression and upregulating superoxide dismutase (SOD) expression [31].

### 2.3. Anti-Inflammatory Activity 

HDL alleviates inflammation by preventing oxidized lipid and lipoprotein formation and facilitating hepatic clearance of oxidized phospholipids and fatty acids [32]. Additionally, it protects against systemic inflammation by clearing circulating endotoxin and serum amyloid-A (SAA) [33,34]. HDL inhibits monocyte adhesion to endothelial cells by downregulating vascular cell adhesion molecule-1 (VCAM-1) and intercellular adhesion molecule-1 (ICAM-1) expression [35,36,37]. HDL also enhances the endothelial barrier and endothelial cell–cell junctions through activation of the S1P/S1P receptor (S1PR) axis, while inhibiting tumor necrosis factor-α (TNF-α)-induced adhesion molecule expression, exerting anti-inflammatory and anti-atherosclerotic effects [38,39]. The protective effects of HDL are mediated by activation of the phosphatidylinositol 3-kinase (PI3K)/protein kinase B (AKT) pathway, suppression of interleukin-1β (IL-1β) and TNF-α release via apo-S1P/S1PR, and inhibition of nuclear factor kappa-B (NFκ-B) nuclear translocation, leading to reduced expression of inflammatory factors and adhesion molecules [40]. Furthermore, HDL reduces monocyte activation by downregulating cluster of differentiation 11b (CD11b) expression [35] and inhibits M1 macrophage polarization through caveolin redistribution, mediated by extracellular signal-regulated kinase 1/2 (ERK1/2) and signal transducer and activator of transcription 3 (STAT3) signaling pathways [41]. HDL inhibits Toll-like receptor 4-induced cytokine expression in macrophages by inducing the transcriptional repressor-activating transcription factor 3 (ATF3) [42].

### 2.4. Endothelial Protection 

HDL has multiple other beneficial physiological functions, such as maintaining endothelial cell barrier integrity, promoting differentiation of endothelial progenitor cells, and inhibiting vascular apoptosis and inflammation [43]. HDL can directly promote endothelial cells to produce nitric oxide (NO) and exert anti-apoptotic and anti-inflammatory effects [44,45]. For example, apolipoprotein E (apoE) 2 has been shown to stimulate endothelial NO release, which enhances vasodilation and prevents vascular inflammation, whereas apoE4 has a pro-inflammatory effect [46]. Normal functional HDL promotes endothelial repair by stimulating endothelial cell migration and proliferation and increasing the number of circulating endothelial progenitor cells mediated by PI3K/Akt-dependent cyclin D1 activation [47]. HDL-associated sphingosine-1-phosphate promotes endothelial progenitor cell proliferation through activation of the STAT3 pathway [48]. Some studies have pointed out that HDL can promote the efflux of 7-oxysterols (such as 7-KC) from endothelial cells through ABCG1, maintain endothelial eNOS activity, and exert endothelial protection [28]. Antioxidant enzymes carried by HDL (such as PON1) can prevent oxidized low-density lipoprotein (ox-LDL) from damaging endothelial cells (ECs) by inhibiting and/or reversing LDL oxidation [25]. In addition to inhibiting LDL oxidation, HDL3 can also improve intracellular antioxidant defense. HDL3 reverses the apoptotic effect of ox-LDL by inhibiting the expression of TNF receptor-associated factor 3 interacting protein 2 (TRAF3IP2) in endothelial cells [31].

### 2.5. Anti-Thrombotic Effects 

HDL can inhibit platelet activation and adhesion by inhibiting thrombotic signaling factors such as tissue factor, P-selectin, E-selectin, platelet-activating factor, and thromboxane A-2, promoting antithrombin expression, changing platelet reactivity, and regulating the coagulation cascade [21,49]. HDL increases nitric oxide production, which inhibits platelet activation and adhesion [44]. Additionally, ox-LDL activates phospholipase A2, promoting platelet aggregation and adhesion. Thus, normal HDL’s reversal and prevention of LDL oxidation may provide protection against ox-LDL-mediated platelet activation [50,51]. Finally, some studies suggest that HDL particles may be involved in fibrinolytic enzyme production, reducing thrombotic risk as well as fibrinolysis [52].

### 2.6. Antidiabetic Activities 

**Figure 2:**
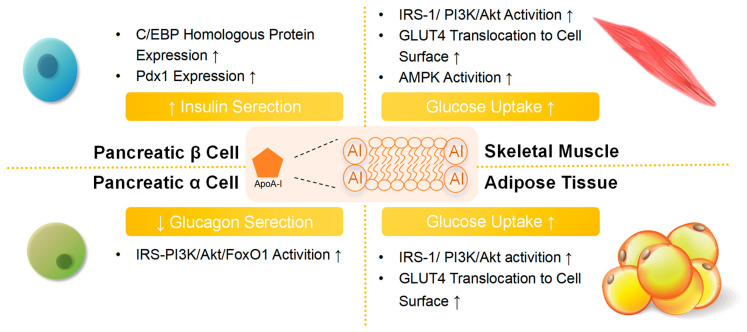
ApoA-I regulates blood sugar. Apolipoprotein A-I (ApoA-I) exerts effects through multiple mechanisms: (i) In skeletal muscle, apoA-I enhances insulin sensitivity by activating the insulin receptor substrate 1 (IRS-1)/phosphatidylinositol 3-kinase (PI3K)/protein kinase B (AKT) signaling pathway, thereby promoting the translocation of glucose transporter type 4 (GLUT4) to the cell membrane. Additionally, AMP-activated protein kinase (AMPK) is implicated in these regulatory processes. (ii) In adipose tissue, apoA-I activates the IRS-1/PI3K/Akt pathway, facilitates GLUT4 translocation to the cell surface, and attenuates inflammation in resident macrophages. (iii) In pancreatic β-cells, apoA-I mitigates β-cell loss and maintains β-cell identity by upregulating pancreatic duodenal homeobox 1 (Pdx1) expression, inhibiting β-cell dedifferentiation into non-insulin-producing cells, and reducing endoplasmic reticulum and oxidative stress. (iv) In pancreatic α-cells, apoA-I suppresses excessive glucagon production by activating the IRS-PI3K/Akt/forkhead box O1 (FoxO1) pathway.

HDL can affect insulin secretion and resistance, improve insulin sensitivity, and thereby affect blood sugar levels through multiple pathways, including stimulating pancreatic β-cells to synthesize and secrete insulin, inhibiting β-cell damage, including apoptosis caused by ox-LDL, and stimulating skeletal muscle, liver and adipose tissue, causing them to absorb and turnover glucose, thereby improving metabolic dysfunction [12,53,54,55,56] (Figure 2). ApoA-I activates the IRS (insulin receptor substrate)-1/PI3K/Akt pathway through ABCA1 and SR-BI, promoting glucose transporter type 4 (GLUT4) translocation to the membrane of skeletal muscle cells and adipocytes, thereby increasing insulin-dependent glucose uptake and improving insulin sensitivity [57,58]. ApoA-I can also enhance the glucose absorption of skeletal muscle cells, enhance β-cell function and peripheral insulin sensitivity through the AMPK (AMP protein kinase) pathway. Metformin enhances glucose uptake and insulin sensitivity in skeletal muscle via AMPK activation, but this effect is absent in *ApoA-I−/−* mice [59,60]. ApoA-I mitigates the detrimental effects of glucotoxicity, lipotoxicity, and glucolipotoxicity by increasing pancreaticoduodenal homeobox 1 (Pdx1) expression in the pancreas and inhibiting C/EBP (CCAAT/enhancer-binding protein) homologous protein, reducing β-cell loss and promoting glucose-stimulated insulin secretion [13,53,61,62]. In the in vitro α-cell model, glucagon was found to serve as an additional therapeutic target for apoA-I and HDL, activating PI3K/Akt/FoxO1 (phosphatidylinositol 3-kinase/Akt/forkhead box O1 pathway) signaling cascade by binding to the cognate receptor SCARB-1 (scavenger receptor class B type 1), thereby regulating glucagon expression and secretion [63]. The above evidence confirms the key regulatory role of HDL in blood sugar and its potential advantages as a new target for diabetes treatment.

### 2.7. Modulating Monocytes and Macrophages and Detoxification 

HDL can also limit monocyte recruitment and restricts macrophage activation and proliferation within atherosclerotic lesions [18]. HDL detoxifies extracellular biohazards through hydrolysis of oxidized phospholipids to directly inactivate harmful molecules on their surfaces (including bacterial lipopolysaccharides and xenobiotics) [64,65].

### 2.8. Kidney Protection 

Changes in HDL levels and function may affect the progression of kidney disease. With the deepening of research on the synthesis and metabolic mechanism of HDL, the kidney is gradually gaining recognition for its significant role in lipid and lipoprotein metabolism. HDL is not metabolized as an intact particle, but as individual components (such as apolipoproteins), contradicting the established concept that HDL exceeds the minimum size of the glomerular filtration barrier and cannot be metabolized by the kidneys [66]. Other cargo of HDL includes proteins involved in lipid metabolism, such as LACT, which are also metabolized by the kidneys [67].

**Figure 3:**
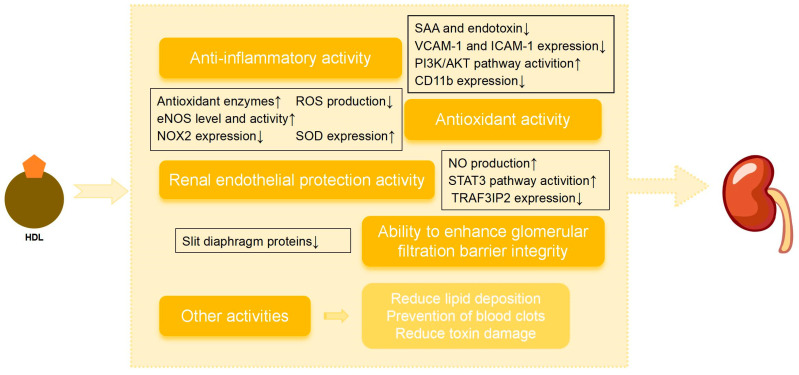
Renal protective function of HDL. HDL and its components can reduce the renal involvement of inflammation, toxic substances and oxidative stress; promote endothelial proliferation and NO production to protect endothelial function and integrity; reduce lipid deposition in the kidneys; prevent thrombosis; and protect the glomeruli and renal tubules through multiple pathways. eNOS: endothelial nitric oxide synthase; SOD: superoxide dismutase; SAA: serum amyloid-A; VCAM-1: vascular cell adhesion molecule-1; ICAM-1: intercellular adhesion molecule-1; PI3K: phosphatidylinositol 3-kinase; AKT: protein kinase B; NO: nitric oxide; STAT3: signal transducer and activator of transcription 3; TRAF3IP2: TNF Receptor-Associated Factor 3 Interacting Protein 2.

Overall, the potential protective action of HDL in DKD can be attributed to several renoprotective activities of HDL: (1) anti-inflammatory activity: HDL has been shown to reduce the production of oxidative products, thereby preventing the activation of the inflammatory cascade and inhibiting local and systemic inflammation [32]; (2) antioxidant activity: the antioxidant activity of HDL prevents oxidative stress-induced cellular damage and inhibits fibrotic processes in DKD [68,69]; (3) renal endothelial protection activity: the HDL particle plays a role in restoring endothelial integrity, thus reducing renal endothelial dysfunction and slowing down the progression of DKD [70]; (4) ability to enhance glomerular filtration barrier integrity: HDL can regulate the glomerular filtration barrier by protecting the structure and function of podocytes and inhibiting the decrease in the expression of slit diaphragm proteins (nephrin and podocin) [68,71]; (5) other activities: HDL inhibits renal microthrombosis, reduces lipid deposition and reduces toxin damage caused by infection. However, it should be appreciated that these protective activities can be adversely influenced or lost when the HDL level is reduced, or its structure and/or components are altered, leading to dysfunctional HDL (Figure 3).

## 3. The Impact of Diabetic Kidney Disease on HDL

CKD is associated with changes in the lipid profile, with DKD being the most common cause of CKD [72]. In most patients with CKD and ESRD, HDL undergoes significant structural and functional alterations. These include markedly reduced HDL-C levels, elevated HDL triglyceride content, and decreased HDL phospholipid composition [73]. Functionally, HDL exhibits impaired antioxidant, anti-inflammatory, and reverse cholesterol transport capacities in these patients [74]. HDL in patients with CKD demonstrates significant oxidative modifications and a pro-inflammatory phenotype when compared with individuals who do not have CKD [27]. Compared to non-diabetic chronic kidney disease, patients with DKD exhibit distinct diabetic HDL-related dyslipidemia characterized by both quantitative and qualitative lipoprotein abnormalities, as well as kinetic alterations, collectively contributing to a pro-atherogenic lipid profile. Qualitatively, HDL in DKD demonstrates increased triglyceride content and enhanced glycation of lipoprotein particles. Kinetically, HDL degradation is accelerated in DKD [75]. These compositional changes in HDL in T2D have been shown to impair HDL functionality, particularly with regard to cholesterol efflux capacity [76]. Furthermore, elevated proteinuria may exacerbate HDL catabolism, leading to increased loss of HDL particles [27].

### 3.1. DKD Affects HDL Levels

With the progression of renal disease, significant alterations occur in the plasma lipid profile; these are marked by a reduction in high-density lipoprotein cholesterol (HDL-C) levels. This occurs as a result of impaired HDL maturation due to reduced apolipoprotein and LCAT [22], abnormal post-translational modifications [77] and slowed hepatic synthesis of apoA-I and A-II [78]. Decreased HDL levels with worsening kidney function and increased levels of the pre-HDL subpopulation that act as lipoprotein lipase inhibitors have been observed in CKD patients [79]. HDL2 and HDL3 levels are reduced in DKD patients, with HDL3 levels declining as CKD becomes more severe [80]. This may be related to impaired LCAT-dependent conversion, as liver LCAT gene expression is downregulated in end-stage renal disease [81].

These abnormalities are also associated with urinary loss of proteins involved in lipid metabolism. Key characteristics of T2D encompass diminished tissue sensitivity to insulin, a condition referred to as insulin resistance (IR), and failure of pancreatic β-cells to secrete sufficient insulin [82]. Enhanced CETP activity in IR and insulin deficiency further promotes CETP-mediated exchange of cholesterol esters and triglycerides in HDL particles [83,84]. Moreover, impaired lipoprotein lipase (LPL) activity under IR condition leads to diminished TG hydrolysis in chylomicrons and very-low-density lipoproteins (VLDL), potentially restricting the formation of TG-enriched HDL particles [85,86]. HDL particles with a lower cholesterol ester/triglyceride ratio are less stable, have increased replacement of apoA-I by SAA, and are more rapidly cleared by the kidney [84]. Elevated HL activity in the IR state may accelerate HDL catabolism and contributes to decreased HDL-C levels [86,87].

However, these lipid abnormalities appear in early renal dysfunction, potentially driven by generalized endothelial dysfunction [88]. Plasma von Willebrand factor (vWF) serves as a key surrogate marker of vascular endothelial injury [89]. vWF is significantly increased in diabetic patients with proteinuria compared with those without proteinuria, which indicates not only kidney damage but also extensive vascular damage [90]. In DKD, endothelial damage elevates vWF and reduces functional endothelial-bound LPL, promoting hypertriglyceridemia and low HDL-C [91].

Structural damage in DKD is often manifested as glomerular sclerosis and tubulointerstitial fibrosis. Disruption of the glomerular filtration barrier and tubular damage may lead to increased numbers and types of HDL particles, apolipoproteins, and enzymes lost in filtration and urine [66]. HDL in T2D patients undergoes methylglyoxal modification, which results in particle reorganization, reduced stability and plasma half-life. This modification also triggers the shedding of smaller HDL particles, which are more susceptible to renal degradation [92]. Albumin plays a role in reverse cholesterol transport by facilitating the transfer of free cholesterol from peripheral tissues to cholesterol-deficient HDL3 particles in the plasma [93]. DKD often presents with prominent albuminuria. Even if the glomerular filtration rate (GFR) is not reduced, proteinuria and hypoalbuminemia can lead to decreased HDL levels [94]. Among type 1 diabetic patients with normoalbuminuria, reduced plasma HDL and HDL3 levels were independently linked to albuminuria even after controlling for glycemic control and other risk factors [95].

### 3.2. DKD Affects HDL Function

Normal apoA-I and HDL in the glomerular filtrate have beneficial effects, stimulating lymphangiogenesis, which promotes uptake and removal of renal interstitial fluid and bioparticles. In contrast, in subjects with dysfunctional HDL, their circulating HDL-C levels are not associated with a beneficial effect, presumably due to a loss or alteration of the HDL functions and a transition to “bad” HDL particles [96].

Compared with non-DKD, the degree of glycation of high-density lipoprotein in DKD is higher, and hyperglycemia leads to an increase in the production of advanced glycation end products (AGE), inhibiting PON-1 activity and the binding of HDL with SR-BI [97]. At the same time, the protein in HDL particles and the reactive alpha-oxoaldehydes (for example, methylglyoxal, glycolaldehyde, and 3-deoxyglucuronide) interact to undergo non-enzymatic glycation [98,99,100,101]. HDL dysfunction can also be caused by reduced HDL surface lipids, defective PON1 activity, and myeloperoxidase-promoted oxidative modification of apoA-I amino acid residues (such as tryptophan, tyrosine, methionine, and lysine) [102,103] Carbamylation, a non-enzymatic post-translational protein modification, is increased in people with diabetes. Carbamylated proteins are implicated in the progression of a variety of chronic diseases, including renal disease [104]. In pathological states, HDL modifications compromise cholesterol efflux, antioxidative capacity, and vasorelaxation; they also diminish the ability of HDL to suppress TNFα-induced NF-κB activation and adhesion molecule expression, to inhibit NADPH oxidase activity and superoxide production, to metabolize erythrocyte membrane hydroperoxides and to exert cytokine-suppressing and anti-apoptotic effects [16,18,105,106].

HDL isolated from diabetic patients exhibits significantly reduced cholesterol efflux capacity (CEC) compared to HDL particles from non-diabetic patients [107]. The reduction in hepatic lipase levels in CKD leads to decreased hydrolysis and clearance of triglycerides in HDL, which in turn enriches HDL triglycerides and reverses the impaired cholesterol transport function [108,109]. In DKD, reduced expression of the cholesterol efflux mediators ABCA1, ABCG1, and apoE correlates with the progression of diabetic nephropathy and deterioration of the estimated glomerular filtration rate (eGFR) [110], suggesting that the development of DKD is associated with reduced HDL-mediated cholesterol efflux [111]. Reduced HDL concentrations, impaired function, and elevated triglyceride levels form a “vicious cycle” that exacerbates dyslipidemia and accelerates the development of atherosclerosis and kidney disease [79].

Under DKD conditions, oxidation and glycation of HDL reduce its anti-inflammatory, antioxidant, anti-thrombotic and endothelium protection ability [112]. Diminished activity of the HDL-associated antioxidant enzyme PON1 and glutathione peroxidase (GPX) can impair the antioxidant activity of HDL [113,114,115]. HDL in chronic kidney disease patients inhibits rather than stimulates NO production, promotes superoxide production, and increases vascular cell adhesion molecule-1 expression [106]. This is because these HDL particles tend to induce phosphorylation of inhibitory sites of eNOS via interaction with the lectin-like oxidizing LDL receptor LOX-1 and the Toll-like receptors TLR2 and TLR4 [116]. These unfavorable receptor-binding properties are acquired as a result of accumulation of oxidized phospholipids, SAA and symmetric dimethylarginine in HDL particles [117]. Elevated SAA levels have been observed in DKD patients. These SAA-rich HDL particles are less anti-inflammatory and even pro-inflammatory, partly due to their acquired ability to increase the secretion of TNF-α from peripheral blood monocytes [118]. HDL isolated from DKD patients is rich in SAA, leading to diminished eNOS activation and impaired endothelial repair [23,119]. Prospective studies have linked high levels of SAA-rich HDL with increased all-cause and cardiovascular mortality, strongly suggesting that SAA modifies the beneficial effects of HDL [120]. HDL particles with increased triglyceride content and reduced cholesterol ester content have pro-inflammatory effects [121]. In DKD condition, enhanced CETP activity promotes the exchange of triglycerides between HDL and other lipoproteins, destroying its anti-inflammatory activity. Increased levels of glycated HDL in DKD impair its capacity to stimulate endothelial cell migration and weaken the vascular protective function [122].

The theory that dysfunctional HDL impairs renal reverse cholesterol transport, promoting intrarenal lipid accumulation, glomerulosclerosis, and tubulointerstitial damage, is well established [78,123]. Dysfunctional HDL affects certain relevant pathways, such as NFκ-B and prospero-related homeobox 1 (Prox-1), in renal tubular epithelial cells and lymphatics, leading to interstitial accumulation of fluid and bioparticles, which can directly cause progressive kidney injury, ultimately leading to a deleterious renal outcome [96]. In addition, since HDL and its components undergo renal metabolism, HDL may directly regulate the renal cells expressing its transporters and receptors, affecting the expression of molecules involved in lipid uptake or efflux, thereby promoting lipid droplet accumulation [124,125], and subsequently leading to extracellular matrix accumulation and fibrosis in the kidney [126]. Lipids can disrupt podocyte function, compromise the glomerular filtration barrier, and alter the synthesis of collagen, laminin and fibronectin, contributing to proteinuria. Accumulation of cholesterol in podocytes plays a role in the pathogenicity of DKD [127]. It appears that the modified apoA-I can be taken up by renal tubular epithelial cells at a higher rate compared with normal apoA-I, a result that increases the likelihood that the deleterious lipoproteins or dysfunctional HDL particles are more readily absorbed and deposited into the renal interstitium, which may exacerbate kidney injury [96]. Significant upregulation of ACAT-1 occurs in the vascular and renal tissues of CKD patients [128]. ACAT-1 catalyzes cholesterol esterification in macrophages and mesangial cells, promoting intracellular retention and foam cell formation. Its regulation in vascular and renal tissues hinders HDL-mediated cholesterol efflux by competing with intracellular cholesterol esterases [129]. In kidney disease, advanced oxidation protein products (AOPPs) (oxidative stress markers carried by oxidized plasma proteins) accumulate in kidney cells and bind to SR-BI with high affinity, blocking HDL from binding to SR-BI, thereby limiting the uptake of cholesterol esters and promoting lipid accumulation [130,131]. Kidney lipid accumulation induces oxidative stress and promotes inflammatory cytokine and growth factor release, accelerating renal disease progression.

Notably, high glucose levels may exacerbate lipid accumulation and damage in the kidneys; for example, a high-fat diet worsens proteinuria and glomerulopathy in diabetic mice, indicating a detrimental concerted effect of lipids and glucose levels on the renal parenchyma [132,133]. Epidemiological and experimental studies support a causal link between dyslipidemia and CKD progression, particularly in patients with diabetes, which amplifies lipid-induced renal damage. Mechanistically, cholesterol homeostasis is closely linked to β-cell function [134]. Impairment of ABCA1- and ABCG1-mediated cholesterol efflux results in elevated β-cell cholesterol levels. This leads to retention of glucokinase in insulin granules and accumulation of cholesterol in mitochondrial membranes, resulting in enhanced mitochondrial stress and β-cell apoptosis, and abnormal insulin secretion [135]. IR may contribute to DKD by driving glomerular hyperfiltration, vascular permeability, subclinical inflammation, and podocyte dysfunction [136,137].

**Figure 4:**
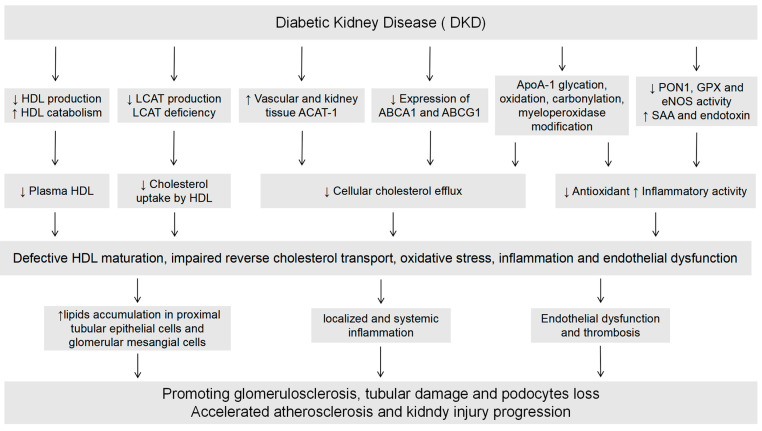
Interaction between DKD and HDL. Diabetic kidney disease (DKD) leads to decreased plasma levels in apolipoprotein A-1 (ApoA-1), apolipoprotein A-2 (ApoA-2), lecithin cholesterol acyltransferase (LCAT) and glutathione peroxidase (GPX), while promoting apoA-I modifications (glycation, oxidation, myeloperoxidase and carbamylation) and increased acyl-CoA: cholesterol acyltransferase 1 (ACAT-1) concentrations in renal and vascular tissues. These changes hinder HDL formation, impair reverse cholesterol transport and exacerbate oxidative stress and inflammation, leading to lipid deposition in the glomerular mesangium and proximal tubules. This promotes glomerulosclerosis and tubular dysfunction and increases the risk of atherosclerosis and DKD progression. PON—paraoxonase; SAA—serum amyloid A.

In summary, the DKD state leads to HDL dysfunction and counteracts the progression of kidney disease. First, damage to the antioxidant and anti-inflammatory properties of HDL may lead to increased oxidative stress and inflammation. Second, endothelial dysfunction and NO deficiency are characteristics of CKD [138,139]. The ability of HDL to promote NO production and maintain endothelial function is essential for maintaining tissue perfusion and preventing leukocyte adhesion and infiltration. Such dysfunction may lead to increased inflammatory cell infiltration in renal tissue. In addition, glomerular mesangial cells can absorb oxidized lipids and lipoproteins, and the proximal tubules can take up filtered apolipoprotein [140]. Impaired HDL-mediated cholesterol transport and antioxidant function drive lipid deposition, promoting glomerulosclerosis, tubular injury, and dysfunction [141,142]. Abnormal HDL function may lead to decreased HDL-bound endotoxin and severe endotoxemia and associated systemic inflammation in patients with ESRD [143], as well as a poor prognosis after microbial infections. Finally, abnormal HDL anti-thrombotic capacity may lead to microvascular thrombosis [144]. Thus, HDL dysfunction may lead to lipid-mediated renal injury (Figure 4).

### 3.3. DKD Affects HDL Components

In addition to the loss of HDL components in the urine resulting in lower circulating HDL levels, the kidneys also regulate HDL components. Experimental proteinuria leads to enrichment of harmful epoxides, diols, hydroxyeicosatetraenoic acid, and hydroxyoctadecadienoic acid in HDL [66,145]; DKD leads to glycation and oxidation of HDL fractions [146]. These changes can significantly alter HDL protein composition, lowering apoA-I, apoA-II, apoAIV, apoE, apoM, and PON-1 levels, while increasing SAA, apoCII, apoCIII, apoD, Lp-PLA2 levels, and surfactant protein B (SP-B), which ultimately reduces their cholesterol efflux and antioxidant capacity [147,148].

ApoC-I and apoC-III are inhibitors of lipolysis and inhibit triglyceride (TG)-rich lipoprotein (TRL) clearance. apoC-III HDL is closely associated with the risk of T2D and reduced insulin sensitivity [149]. In particular, TRL-apoC-III levels are significantly increased in DKD [150], and the molecular mechanism responsible for apoC-III upregulation is unclear. ApoA-V levels were significantly reduced in diabetic hemodialysis (HD) patients [151]. Although the plasma concentration of ApoA-V is very low, it may serve as a potential regulator of plasma TG and plays an essential role in the development of hypertriglyceridemia in some HD populations [152]. In addition to the main components of apolipoproteins, more and more low-load protein and lipid components are being studied for their effects on dyslipidemia, which will further help to identify new disease predictors and therapeutic targets.

## 4. U-Shaped Relationship Between DKD and HDL-C Level

HDL levels are a significant marker that predicts the development and progression of DKD [153]. Low HDL-C levels (especially decreased serum apoA-I and apoA-II concentrations) are associated with the incidence of DKD, which may be due to the effects of HDL on pancreatic β cells and vascular endothelial cells [56,154]. Low HDL-C levels reduce antidiabetic and kidney protection, and increase oxidative stress and inflammation, which are key factors in the pathogenesis of renal damage in diabetic patients [155].

Because the kidneys filter, reabsorb, catabolize and excrete apoA-I and other components of HDL, low circulating apoA-I or HDL levels may reflect generalized renal dysfunction before other established markers such as reduced glomerular filtration rate or increased proteinuria [66]. Low apoA-I or HDL may be a sign of early/mild disruption of the glomerular filtration barrier and/or tubular reabsorption capacity [66]. Epidemiological studies show a negative association between plasma HDL-C levels and the risk of developing T2D, with HDL-C serving as an independent predictor of diabetic complications [156,157,158]. Mendelian randomization studies also suggest a negative correlation between HDL-C and T2D risk [159,160], and high HDL-C levels potentially offer protection against DKD [161].

The HDL-C concentration observed during the development of T2D-DKD may vary by disease stage and gender. A cohort study involving T2D patients with or without albuminuria suggests that diminished HDL-C levels are linked to the progression of proteinuria in men, but not in women [162]. Another study by Yadegar et al. reported that a decrease in HDL-C levels in patients with T2D-DKD is associated with CKD stage progression [163]. Furthermore, low HDL-C levels may reflect dyslipidemia, including elevated total cholesterol (TC) and LDL-C, which heighten vascular damage risk and accelerate diabetic microvascular complications.

However, at a time when a large number of studies have supported, from an epidemiological perspective, the protective effects of HDL, including on cardiovascular disease and kidney disease, with HDL being viewed as “good cholesterol”, the idea emerges that excessive HDL levels may increase the risk of cardiovascular events [164,165,166]. An association between very high HDL-C (primarily defined as >100 mg/dL) and diabetes has been demonstrated [167]. A U-shaped relationship between HDL-C levels and renal outcomes has been established, with both low and high HDL potentially accelerating CKD progression, though the specific determinants remain unclear [15,168]. The prevalence of proteinuria in diabetic patients was associated with HDL ≥ 100 or ≤40 mg/dL, and this association was strengthened after adjusting for body mass index [169]. Some studies have also pointed out that 0.95 and 1.54 mmol/L are selected as the thresholds for DKD risk in T2D patients. After adjusting for confounding factors, the DKD risk of T2D patients in the high-HDL-level group is even greater than in those with low HDL levels [170]. However, due to differences in the selection of research populations and measurement methods, the specific threshold of protective HDL-C has not been unified. Interestingly, this nonlinear relationship was also found to be gender-specific, with a more pronounced effect in women, while it was not statistically significant in men [170]. This may be related to gender differences in blood lipids, and indeed, estrogen can promote an increase in HDL [171,172]. These findings imply that at elevated concentrations, HDL-C may lose its protective effects.

The mechanisms supporting this observation are not fully understood. Experimental evidence suggests a biphasic effect of HDL-C (at both low and high concentrations), with elevated HDL-C levels potentially accelerating senescence and impairing endothelial progenitor cell function, limiting tube formation and angiogenesis, thereby indicating a loss of protective effects at high HDL-C levels [173]. In the setting of high C-reactive protein levels, the beneficial effects of high HDL-C concentrations are reversed, and inflammatory cytokine activation and increased inflammation may be the key risk factors for CKD progression in subjects with elevated HDL-C [164,174]. Current dyslipidemia studies often focus on the level of HDL-C, but it should be noted that patients with very high HDL-C levels may also have very high levels of LDL-C and other supposedly “bad” lipids, as well as total cholesterol, which may overwhelm and deplete the protective effects of HDL, thus creating the illusion of HDL-induced damage [175].

Increased HDL levels are not necessarily parallel with enhanced HDL function. When HDL levels rise, the proportion of HDL subfractions with specific protective activities may decrease, which simply increases inactive or dysfunctional HDL particles, leading to further negative effects [176]. At the same time, high concentrations of HDL-C have impaired functions, including reduced reverse cholesterol transport and anti-inflammatory activity, which may increase the risk of renal complications [177]. Genetic factors may play an important role in this complex relationship [178]. Therefore, it is important to pay attention not only to the concentration of HDL, especially when receiving HDL treatment, but also to whether its function has changed [179].

Furthermore, in a diabetic environment that results in genetic variations and potential changes in conformational and functional properties of genes such as CETP, ABCA1, SRBI, HL, and SCARB-1, high HDL levels may exacerbate the damage caused by aberrant pathway expression [76,180]. In the diabetic state, the U-shaped curve between high HDL-C levels and all-cause death is more typical, which also confirms that the clinical importance of HDL-C increases as the glycemic state worsens [180].

In general, the risk of DKD associated with excessive HDL-C concentrations may be related to a decrease in the proportion of beneficial HDL components and a simultaneous increase in “bad” blood lipid components. In addition, hyperglycemia induces abnormal HDL modifications, disrupting its function and action pathways, thereby impairing its anti-inflammatory, antioxidant, and anti-atherosclerotic properties, which contribute to microvascular disease and renal dysfunction. Elevated circulating HDL-C promotes its accumulation in the kidneys and other parts. When dysfunctional HDL particles and the pro-inflammatory particles they carry (such as SAA) accumulate in the kidneys, they will induce changes in renal structure and damage renal function. Further research is required to elucidate the underlying mechanisms

## 5. The Potential of HDL as a Clinical Diagnostic and Therapeutic Target for Diabetic Kidney Disease

### 5.1. HDL as a Risk Biomarker for Development of DKD

Identifying potential lipid markers that influence the onset and progression of DKD would be useful in preventing and/or slowing the advancement of DKD to ESRD. In addition to the single HDL-C circulating concentration, more HDL-related indicators have been identified as potential predictors of disease risk.

High TG/HDL-C ratios are considered to have detrimental effects, causing endothelial dysfunction, chronic low-grade inflammation, increased oxidative stress and abnormalities in fibrinolysis and coagulation [181,182]. Elevated TG/HDL-C ratios, commonly seen in diabetic and CKD patients, are associated with increased insulin resistance and atherogenicity [183]. These results have also been validated in Japanese and Italian populations, where the TG/HDL-C ratio was positively linked to the development of DKD, independent of other variables (e.g., smoking, hypertension, etc.) [182,184].

HDL2-C exhibits a stronger negative correlation with body weight, fasting glucose and insulin, 2-h glucose, insulin resistance index, and C-reactive protein than HDL-C [185]. Diabetic patients receiving insulin therapy have higher HDL2-C levels than those taking oral medications. HDL2-C has been considered “metabolic HDL” and is being studied more as an indicator of insulin resistance [186]. Lower HDL2-C is a marker of microalbuminuria in patients with type 1 diabetes [187].

The non-HDL-C/HDL-C ratio (NHHR) is an emerging lipid marker capable of predicting diabetes risk and its complications [188]. ApoM is a member of the lipid-transporting protein family and plays a key role in the formation of pre-β-HDL particles [189]. Plasma apoM concentrations are significantly higher in DKD patients compared with non-DKD patients [189]. The apoM/HDL-C and apoM/apoA-I ratios are predictors for DKD development in both healthy controls and T2D patients [189].

Higher lipid variability (changes in lipid levels over multiple occasions) has also been shown to be associated with poorer health outcomes in diabetic populations [190,191]. Population studies have found that the greater variability in LDL, HDL and TC in people with T2D is associated with a significantly increased risk of death and may lead to an increased risk of developing microvascular complications of diabetes, such as DKD [192].

Kidney tubular epithelial cells express apolipoprotein receptors (cubilin/megalin) and cholesterol transporters (ABCA1 and SRBI), which can internalize and degrade apoA-I [193]. Therefore, glomeruli and renal tubules can filter, absorb, degrade or recycle apoA-I, returning it to the circulation and regulating plasma apoA-I and HDL levels. A parallel relationship between glomerular filtration barrier disruption and urinary excretion of apoA-I was found in mouse models of podocyte injury with/without proteinuria, and could reflect glomerular filtration barrier disruption more sensitively than albumin [66]. Similarly to apoA-I, apoA-IV levels are affected by glomerular filtration and tubular reabsorption and catabolism. In humans, urinary apoA-IV and LCAT are elevated in the early stages of kidney injury and have been considered markers of CKD, and increases in plasma apoA-IV when the glomerular filtration rate decreases may reflect reduced kidney clearance [67,194]. It is postulated that the development of urine markers may be useful for further comprehensive evaluations of dyslipidemia and renal damage.

### 5.2. Therapeutic Strategies to Enhance Levels and Function of HDL in DKD

Given the important role of HDL in cardiovascular and metabolic health, there has been widespread interest in developing HDL-based therapies. In T2D, HDL particles with high LDL/HDL ratio and low levels of apoA-I are associated with DKD [195]. Therefore, strategies to raise HDL-C level are potentially useful to provide renal benefit.

For example, niacin and fibrates were developed to lower plasma TG and significantly increase circulating levels of HDL-C [196]. Omega-3 fatty acid can also decrease TG, but has no significant effect on HDL [197]. Statins, ezetimibe, and PCSK9 inhibitors can slightly increase the plasma levels of HDL-C, but their cardiovascular and renal benefits are only causally related to their ability to primarily lower LDL-C [196,198,199,200].

Among the large randomized controlled trials with T2D patients, the FIELD and ACCORD studies found that fenofibrate, a drug that lowers TG and LDL-C by increasing lipolysis, increased serum HDL levels and reduced the incidence of microalbuminuria and macroalbuminuria compared to the placebo group [201,202]. Renoprotective effects of fenofibrate with reduced progression of albumin excretion compared with placebo were also seen in The Diabetes Atherosclerosis Intervention Study (DAIS) [203,204].

Niacin is a B vitamin that stimulates the production of apoA-I in the liver, thereby increasing HDL levels [205]. Myeloperoxidase (MPO) is involved in reactive oxygen species-induced tissue damage and the production of dysfunctional HDL [206,207], and can promote oxidative stress, inflammation, and endothelial dysfunction leading to the progression of chronic kidney disease [208]. MPO is elevated and significantly correlated with the albumin–creatinine ratio in DKD patients [209,210]. Niacin can significantly reduce the release of MPO from leukocytes, improve endothelial dysfunction, and prevent HDL dysfunction [211,212]. The renal protective effect of niacin treatment has been confirmed in animal experiments [213], but its effects and mechanisms on human kidneys are still controversial [214,215].

Administration of exogenous HDL via infusion is another possible approach to enhance the beneficial effects of HDL. Based on evidence from preclinical studies indicating that an intravenous HDL infusion reverses atherosclerotic plaque, efforts have been made to develop reconstituted HDL (rHDL), which has been shown to specifically enhance HDL-mediated reverse cholesterol transport (RCT) [216]. ApoA-I has been shown to be a potential therapeutic option to improve insulin sensitivity [57,58]. Reversal of impaired glucose tolerance and a reduction in fasting blood glucose levels was achieved as a result of a single infusion of apoA-I in animal models and T2D patients [217]. Multiple HDL mimetics have been developed and tested in rodents and in humans [44].

ApoA-I analog peptides L-4F and RG54I were shown to improve insulin resistance and glycemic control by increasing hepatic insulin receptor expression and glucose uptake by skeletal muscle myotubes in fat-fed C57BL6 and db/db mice [218,219,220]. L-4F was found to improve the glomerular filtration rate, reduce tubular injury and protect renal function in a cecum ligation puncture (CLP) rat model in an HDL-dependent manner [221]. Recent studies have shown that treatment with rHDL containing apoA-I as the main component improves kidney histology and function in multiple diabetic mouse models and can slow down the progression of DKD, specifically, with improvement in proteinuria, prevention of GFR decline, attenuation of glomerular tunica dilatation/glomerulosclerosis, ECM protein accumulation and renal fibrosis as well as inhibition of podocyte loss [222,223,224].

ApoA-IMilano, a natural variant of apoA-I, enhances HDL cholesterol efflux capacity compared to the wild type [225]. This led to the development of recombinant HDL (MDCO-216) containing purified ApoA-IMilano and phospholipids; however, trials were discontinued due to a lack of efficacy [226,227].

A CEPT inhibitor can increase plasma HDL-C and apoA-I levels in humans, with improved glycemic control as a result of enhanced pancreatic β cell function and increased insulin sensitivity [228,229]. A follow-up analysis of a large clinical trial of the CETP inhibitor highlights an antidiabetic property of HDL [230,231]. This effect was also reproduced using other CETP inhibitors, such as torcetrapib and evacetrapib, in two independent studies in T2D cohorts [232]. A meta-analysis of all CETP inhibitor trials showed a 12% reduction in the incidence of T2D [38,233]. CETP inhibitors improve glycemic control in T2D patients and lower new-onset diabetes risk in non-diabetic individuals compared to placebo [234]. The renal effect of CETP inhibitors is currently unknown, since there are no population-based studies of CETP inhibitors with renal outcome as a key endpoint.

These above findings postulate that normal apoA-I and apoA-I mimetics with certain beneficial functions, such as antidiabetic and anti-inflammatory activities, could be nephroprotective, but this remains unproven in studies focused on humans. There are a number of ways to enhance the beneficial functions of rHDL particles, such as using Apo A-I variants with greater protective activity, optimization of the lipid composition and inclusion of antioxidants, and/or certain micro-RNAs (miRNAs). The administration route may be able to influence the behavior of the rHDL, which should also be optimized to maximize the beneficial effects of rHDL.

Circulating HDL functions as an endogenous nanocarrier, delivering proteins, vitamins, hormones, and microRNAs to various organs [235]. Furthermore, the generation of HDL-based nanoparticles is a new research direction, where HDL particles can be used as drug carriers to deliver specific therapeutic agent(s) to target certain organs or disease sites [236]. Nanodisk (ND) technology formulates disc-shaped rHDL, incorporating non-native hydrophobic bioactive molecules for targeted delivery [237]. Properly synthesized NDs retain the solubility, structure, and stability of classical rHDL while exhibiting unique properties from their non-physiological components [237]. It has been reported that endogenous HDL is involved in the transport of microRNA in vivo, which suggests that HDL may be a natural transport carrier for nucleic acids [238]. Compared with other kidney disease patients and healthy people, the level of miRNA-181b-5p in the serum of DKD patients is lower, and animal experiments have confirmed that supplementation with miRNA-181b-5p mimics reduces albuminuria and abnormal mesangial expansion in DKD mice [239]. The development of HDL-related transport carriers will help expand the application scope of microRNA as a therapeutic target for DKD. This strategy combines the rHDL-inherited property of tissue/organ protection and the specific therapeutic effects of the delivered drug(s). Such an approach may help to slow the progression of diseases.

In addition to their primary role as glucose-lowering agents, certain antidiabetic drugs have demonstrated potential lipid-modulating effects in the context of DKD. A systematic meta-analysis of Sodium-Glucose Cotransporter 2 Inhibitors (SGLT2is) has revealed that these agents significantly elevate HDL-C levels while reducing TG concentrations [240]. This lipid-modulating effect may be attributed to improvements in insulin sensitivity and enhanced insulin secretion [241]. Clinical trials have further demonstrated that dapagliflozin, a representative SGLT2i, influences HDL-C levels and modifies lipoprotein size and functionality in T2D patients, although no significant intergroup differences were observed in another study after adjusting for confounding factors such as age and body weight [242]. Notably, another study reported a specific increase in large HDL2 subfractions among diabetic patients treated with dapagliflozin [243]. To further elucidate the impact of SGLT2is on HDL functionality, prospective randomized clinical trials are currently underway [244]. The effects of other glucose-lowering agents, including metformin [245], glucagon-like peptide-1 (GLP-1) receptor agonists [246,247], and dipeptidyl peptidase-4 (DPP-4) inhibitors [248,249], on HDL levels and functionality remain inconclusive and warrant further investigation.

Overall, increasing serum HDL-C levels or augmenting HDL components may have beneficial effects on T2D and DKD. Lifestyle changes (such as modest weight loss, increased physical activity, and smoking cessation when appropriate) are known to be able to increase circulating HDL and apo A-I levels, to lower the TG/HDL-C ratio, and to reduce diabetic microvascular complications. Secondly, a variety of lipid-regulating drugs targeting lipid metabolic pathways have been developed, further broadening the HDL-related therapeutic window, but the protective mechanisms that could influence certain diseases needs to be studied in more detail. The beneficial effects of simply increasing HDL levels are questionable, so the rHDL method, which can specifically synthesize the effective components of HDL, has been proposed. The renal protective effects of rHDL and its potential additional role as a carrier of antidiabetic drugs provide promising treatment options for DKD patients. Future studies should focus on optimization of the composition and delivery method of HDL particles and exploration of new areas of HDL-targeted therapy. In addition, the impact of some demographic factors such as gender and race on HDL-related treatments should be more robustly investigated in order to provide evidence-based selection of patients who would benefit from receiving HDL-associated treatments.

## 6. Conclusions

HDL is a biological particle that mediates reverse cholesterol transport to remove and clear excess lipids from cells, leading to anti-atherosclerotic, antioxidant, anti-inflammatory and anti-thrombotic effects. These beneficial properties of HDL have been postulated to promote and maintain cardiovascular and metabolic health. HDL level, composition and structure can be altered in patients with diabetic kidney disease, leading to impaired HDL function and thus further impairing glucose homeostasis and kidney health. Low HDL-C levels are a significant risk factor for DKD, with dysfunctional HDL particles identified in patients with this condition. In patients with very high HDL-C levels, the observed increases in the progression of diabetic kidney disease and all-cause mortality have indicated that HDL-C may play an adverse role when its levels are excessively increased. This may be because the elevated HDL is mostly composed of dysfunctional particles, and abnormalities in multiple blood lipid components and lipid deposition cause additional renal and systemic damage. Therefore, we believe that when HDL functions normally, it can counteract hyperglycemic damage to the kidneys and other organs and delay the development of diabetic kidney disease. However, as the disease progresses, some HDL modifications increase, and its protective effect is reversed, becoming a risk factor. At this time, its comprehensive effect cannot be evaluated simply by HDL levels, and specific functions should be measured to determine whether it is a friend or a foe.

The antidiabetic and vasoprotective effects of HDL indicate that improving HDL function may be a promising strategy for preventing and treating diabetes and its complications, including DKD. Future research on developing HDL pharmacomodulation should focus on functional optimization of HDL particles by carefully engineering the composition and other properties of the HDL products, including using the HDL particle as a delivery vehicle for additional therapeutic agents, rather than just simply increasing the HDL level.

## Data Availability

No new data were created or analyzed in this study. Data sharing is not applicable to this article.
